# Dr. Kinglake, on the Practice of Blood-Letting

**Published:** 1808-11

**Authors:** Robert Kinglake

**Affiliations:** Taunton


### *To the Editors of the Medical and Physical Journal.*

GENTLEMEN,

AMPLE experience has convinced me of the vast importance of correctly directing the practice of blood-letting, in the relief and cure of various states and conditions of disease. The general advantages of this remedy, when indicated by indubitable signs of systematic strength, vascular fullness, and immoderate excitement, are too evident to be at all questionable; it is when the seeming occasion for its employ is rendered doubtful by the presence of symptoms that would appear to contra-indicate its adoption, that it is of moment to resort to it in such a manner as may prove salutary, without incurring any risk of doing injury.

In this neighbourhood, a pulmonic affection has lately prevailed, bearing a strong resemblance to that character of disease which has been denominated Peripneumonia Notha. In this complaint, respiration is often much embarrassed, accompanied with a short dry cough, and more or less of pain in the region of the thorax, sensibly augmented by deep inspiration; the pulse is usually full and quick, but, in most instances, has but little or nothing of that tensive hardness, which denotes the existence of inflammatory affection; the tongue is dry and coated; thirst urgent; the skin hot and unperspiring; and febrile chilliness and heat alternately occur at irregular intervals. The disease which is announced by this groupe of symptoms, is that which has deservedly induced great caution with regard to bleeding. Instances certainly abound of irreparable mischief having resulted from an unguarded loss of blood in the recited affection. The evacuation has been often followed by a rapid declension of strength, and a speedy termination of life. On the contrary, it must be confessed, the disease has been known to advance in its destructive career, when bleeding has not been resorted to, but when a tonic and sustaining mode of treatment has been adopted.

The experienced medical practitioner, must have seen enough of this disease, to be persuaded that bleeding in its treatment, is rather a remedy to be occassionally employed with discriminating caution, than to be wholly withheld. The thoracic difficulties presenting in this disease are often much relieved by the loss of not more than three ounces of blood: that small quantity removes the morbid fullness of the vessels, usually existing on these occasions, without inducing additional weakness; it also tends to facilitate the transmission of the circulating fluids, to promote a determination to the surface, and thus to render essential benefit. Three ounces of blood, drawn at any stage of this disease when respiration is much oppressed, will be found sensibly to alleviate the pressure of that symptom, when the most extensive blistering of the chest will not avail; and it will often occur, that external irritation, either by vesication, pustulation, or rubefacience, will be additionally efficacious in relieving strictured and painful breathing, when preceded by a moderate loss of blood.

The great practical end obtained in proposing the removal of so small a quantity of blood, as cannot sensibly diminish strength, whilst it directly and permanently lessens the patient's suffering, is, to avoid the danger of what has been held as a doubtful remedy, by employing it in such a manner as may prove salutary, without the possibility even of its being injurious.

The

The indication for small bleedings is not confined to peripneumonia notha, it occasionally extends to every variety of complaint connected with an undue degree of vascular distention. It is indeed highly gratifying to observe, how much the stimulant influence of vascular plenitude is relieved by a small evacuation of blood. Striking examples of this are afforded in the menstrual and hæmorrhoidal discharges, and also in periodical bleedings from the nose; on these several occasions, painful distention is felt either in some particular viscus, or throughout the system at large; the quantity of blood that escapes in these cases, rarely exceeds three or four ounces, and yet this is found sufficient to restore the morbidly distended vessels to the healthful state.

The stimulus of distention is the most universal, and the most efficient that the animal economy is subject to; the functions of both life and health are connected with the laws of its action; it is, therefore, of the utmost consequence, that a due regard should be had to its influence in considering the nature and cure of diseases. The various operation of emetic, cathartic, sudorific, and diuretic medicine, respectively goes to abstract from the vascular system a portion of its contents, by which hurtful distention is relieved, and any immoderate action depending on that state is likely to be subdued. Hence it appears, that the occasion for diminishing the circulating fluids is co-extended with that of excessive action and fullness of vessel, yet it is more especially necessary in relieving the pressure of symptoms arising from an interruption given to the passage of the venous blood from the *vena cava* through the lungs to the heart. Whenever this occurs, whether in peripneumonia notha, in apoplexy, in epilepsy, in the hot stage of fever, or in suspended animation from drowning and other causes, a loss of blood proportioned in quantity to the circumstances of the case is indispensably requisite.

Morbid action arising in any part of the body may awaken a degree of general excitement, that may painfully distend the vascular system, and induce a necessity for diminishing the circulating fluids, either by blood-letting, or by some increased secretion. In phthisis pulmonalis, small bleedings, as indicated by occurring pain or oppression on the lungs, are highly beneficial. A patient under my direction, who ultimately died of tuberculated lungs, as ascertained by dissection, survived upwards of two years what was considered as the last stage of pulmonary con-

 sumption, sumption, manifestly by the aid of small and repeated bleeding: not more than from two to three ounces were ever taken at once; and the urgency of thoracic oppression, approaching even to actual suffocation, absolutely required that it should be resorted to, as the *unicum remedium*, every three or four days. A well regulated course of bleeding, in the early stage of phthisis pulmonalis, appears to me, to afford more unequivocal benefit than any other mode of assistance. By relieving vascular distention, it improves the pulse, and contributes to protect and eke out the systematic strength, rather than to impair and hasten its demolition.

Advantageous as small and well-timed bleeding actually is, and may be, in various states of disease, yet the observant practitioner must have borne painful testimony to the irreparable mischief it is capable of producing, when indiscriminately and unjustifiably employed. An unhappy prejudice seems to have long had a baneful currency in medical practice, respecting a warranty for repeated blood-letting derived from the *aspect* of the blood drawn. It is confidently held, that what is commonly termed the buffy or yellowish appearance at its surface, indicates the existence of inflammatory action; and it is farther insisted, that the quantity of blood necessary to be evacuated in that state, may be safely and salutarily measured by the continuance of that appearance.

This opinion appears to have gained general assent, and is dogmatically upheld with all the authority due to an indisputable axiom. When it is considered, that the appearance on which so much practical reliance is placed is fallacious, in as far as it is more the result of accident in the process of coagulation, than a sure criterion by which to judge of any condition of either vascular action, or vascular fluid requiring the removal of blood, it must be obvious that irretrievable injury must have been often occasioned by this prevailing misapprehension. It is well known, that if blood be drawn from a large orifice, and received in a deep and narrow vessel, such as a tea cup, it will almost, under any circumstances of either health or disease, exhibit on its coagulated surface the buffy aspect under consideration. This effect necessarily results from the contracted surface exposed to the atmosphere causing coagulation to obtain slowly, and thus affording the globular or red part of the blood time to subside, leaving the coagulable lymph, with which it was previously mixed, on the surface. If the same blood were received into a tea *saucer*, instead of a tea *cup*, the ensuing coagulation would speedily ly take place, and obviate the separation and consequent subsidence of the red particles from the coagulable lymph; hence, the aspect would be florid, and the accredited yellow sign of inflamed blood would be wanting; a circumstance therefore so accidental as the aspect of drawn blood generally is, deserves not to be considered as a valid authority for blood-letting, in diseases in which that remedy, without such an appearance, would be deemed questionable.

To persist in drawing blood as long as its surface shall exhibit a buffy aspect, has been the *sanguinary* (to use no stronger epithet) direction of those who have implicitly depended on the supposed sure guide, afforded by this casual and unimportant appearance. It has occurred to me, to observe much of the destructive influence of this inconsiderate practice, and to have, in many instances, exposed the deception that had warranted it, by requiring that the suspicious blood should be received in a superficial, instead of a deep vessel.

The stimulant effect of extreme debility, as well as that of excessive tone, very generally induces more or less of painful sensation, which would be very inaptly and injudiciously treated by blood-letting; yet the aspect of blood drawn in such a state, might, if received in a deep bason, in the accustomed manner, be of the buffy description, which, agreeably to popular opinion, would at once serve to determine the inflammatory nature of the disease, and demand repeated evacuations of blood. Against this heedless and pernicious practice, it behoves every person, conversant with its hurtful effects, to enter a decided protest, and to declare it to be neither authorised by any rational principle of physiology, nor by correct experience.

The texture of drawn blood is a less fallible test of the state of vital action, than that which is afforded by its superficial colour; when dense and cohesive, the existence of an adequate degree of vascular tone and energy is evinced; on the contrary, when loose and easily separable, it may be justly considered as the effect of deficient strength.

These different conditions, respectively, furnish appropriate indications of cure; in the one case, a reduction of vital power; in the other, its augmentation and support; on the former occasion, blood-letting is a direct and powerful remedy; on the latter, it would be necessarily injurious, and may prove destructive.

I am, &c.

ROBERT KINGLAKE.

*Taunton, Sept.* 17, 1808.

